# Development of a Method for Detecting and Estimating *Moniliophthora roreri* Spore Loads Based on Spore Traps and qPCR

**DOI:** 10.3390/jof9010047

**Published:** 2022-12-28

**Authors:** Diana L. Jiménez-Zapata, Manuela Quiroga-Pérez, Manuela Quiroz-Yepes, Alejandro Marulanda-Tobón, Javier C. Álvarez, Sandra Mosquera-López

**Affiliations:** 1 Division of Natural Systems and Sustainability, School of Applied Sciences and Engineering, EAFIT University, Medellín 050021, Colombia; 2 CIBIOP Research Group, School of Applied Sciences and Engineering, EAFIT University, Medellín 050021, Colombia; 3 GEMA Research Group, School of Applied Sciences and Engineering, EAFIT University, Medellín 050021, Colombia

**Keywords:** spore trap, qPCR, moniliasis, *Theobroma cacao*, frosty pod rot

## Abstract

Frosty pod rot, caused by *Moniliophthora roreri*, is the most damaging disease of cacao in Latin America and, to better comprehend its epidemiology, we must understand its dissemination and proliferation. However, we do not know how *M. roreri* spores loads fluctuate in time and space due to the lack of a reliable technique to quantify *M. roreri* spores in the fields. Therefore, we developed a method that relies on spore traps and qPCR to detect and quantify *M. roreri* spore loads. This study demonstrated that the qPCR protocol can detect down to 0.025 ng of *M. roreri* DNA and quantify between 0.006 ng and 60 ng. Moreover, it demonstrated that qPCR protocol can detect and quantify DNA extracted from spore suspension and spore traps containing at least 2.9 × 10^4^ *M. roreri* spores. However, the variability of the estimates for spore samples was high. Finally, we described a spore-trap device designed to carry spore traps in the field. The qPCR protocol and spore-trap device here developed will help in the understanding of the *M. roreri* dissemination patterns since they can be used to assess the environmental loads of *M. roreri* spore in cacao fields.

## 1. Introduction

Frosty pod rot (FPR), caused by the basidiomycete *Moniliophthora roreri,* is the most prevalent and severe cacao disease in Latin America [1]. This disease causes production losses between 16% and 100% depending on the growing region and agronomic factors [1,2,3]. In Colombia, the production losses derived from FPR are close to 40% but can be 100% in some plantations [4]. FPR exclusively affects the cacao pods, beginning as chlorotic spots on the pod’s exterior, which turn dark brown as the disease progress. In severe diseases, brown spots grow and cover the pods’ surface. Then, pods turn white and powdery as *M. roreri* mycelia and spores become visible [2,5]. Spore-covered pods can carry about seven billion spores, each capable of initiating a new infection. Understanding how *M. roreri* spores disseminate is critical for the pathogen’s epidemiology and designing control strategies to mitigate FPR losses [6].

Only a handful of *M. roreri* studies have evaluated the spore dissemination in the field or the environmental spore loads, and their results are mostly inconclusive [2,5,6,7,8,9]. Still, we have gained some insights. The literature shows that *M. roreri* environmental spore loads vary in time and space and that climatic variables influence them at different scales [3,5,6,8]. On a small scale, mist, wind, and rain are critical since they move *M. roreri* spores away from the inoculum source (i.e., sporulated pod) [10]. Multiple evaluations have shown that FPR incidences and M. *roreri* spore loads decrease as they move away from the inoculum source until they plateau and remain at low levels [5,7]. These low levels of spore loads are critical for *M. roreri* disease cycle since they constitute a background inoculum, always present in cacao plantations and ready to initiate an FPR infection [4,5,7]. The distance from the inoculum source at which *M. roreri* spore loads plateau is unclear. Some studies suggest that spores can travel more than one kilometer without reaching this plateau, while others indicate distances below 375 m [4,5,7]. It is also unknown whether the spore loads will eventually drop to zero at farther distances.

Inconsistencies are also found in studies addressing the *M. roreri* spore dissemination and environmental spore loads at larger scales. For example, two studies evaluating the daily spore load variation found that the highest concentration of *M. roreri* spores occurred around noon [5,8], while another found that it occurred at night [11]. On a larger temporal scale, some studies have found that dry months with relatively high temperatures (close to 30 °C) favor the long-distance spread of the pathogen and relate to increased spore loads in the environment [2,5,8,9,11]. However, this association was weak since it was supported by noisy data [5,8]. 

The inconsistencies in the *M. roreri* literature regarding spore dissemination and environmental spore loads might come from the use of outdated methods. Half of the evaluations are nearly three decades old [5,7,8], and most used spore traps and microscopy to collect, identify and quantify spores [5,8,11]. This approach might be appropriate for some fungal pathogens [12]. However, *M. roreri* spores have variable shapes and sizes due to their conidiogenetic process [11,13]. Therefore, using microscopy to identify and quantify them can lead to errors. An alternative to microscopy is to grow spores in spore traps in culture media and quantify colonies [14]. *M. roreri* colonies can take several weeks to develop and there is no culture media specific to *M. roreri.* Therefore, growing trapped spores in culture media is also not feasible for *M. roreri* since it is time consuming and colonies can be overgrown by other fungi in the environmental samples [15]. 

Quantitative PCR (qPCR) is a fast and reliable alternative to microscopy and colony culture. Several studies have successfully coupled spore traps with qPCR to analyze the environmental spore loads of several plant pathogens [16,17,18,19], but none have used it for *M. roreri*. This study aimed to develop a technique coupling spore traps with qPCR for detecting and quantifying *M. roreri* spores. Such a technique could facilitate future studies evaluating the environmental *M. roreri* spore loads in cacao plantations. During this study, we developed a qPCR protocol specific to *M. roreri*, which was validated following the MIQE Guidelines [20]. Then, we used this protocol to detect and quantify *M. roreri* spores in suspensions and spore traps. Finally, we describe a spore trap device designed for carrying the spore traps in the cacao fields. The qPCR protocol and spore trap device here develop are useful for the understanding of the *M. roreri* epidemiology since they will be used in further evaluations to asses the *M. roreri* spore loads in cacao plantations with FPR prevalence.

## 2. Material and Methods

### 2.1. Fungal Strains and Cultures

The *M. roreri* strains used in this study were isolated from cocoa pods with late FPR symptoms collected from commercial farms in representative cacao-growing regions in Colombia (Table S1). Specifically, we hit the symptomatic pods over ×0.5 potato dextrose agar PDA (OXOID, Cheshire, UK) plates supplemented with kanamycin (Thermo Fisher Scientific, Boston, MA, USA) at 50 μg/mL to release *M. roreri* spores. Plates were incubated at 30 °C for three days. Single-germinated spores were transferred to plates containing malt extract agar (MEA) (OXOID, Cheshire, UK) using a needle and a stereomicroscope Discovery V12 (Zeiss, Germany). Then, they were incubated at 30 °C for nearly one month (the time required by the colonies to grow and sporulate). Spores were washed off MEA plates with sterile 20% glycerol (Thermo Fisher Scientific, Boston, MA, USA) and stored at −80 °C as 20% glycerol stocks until needed.

We attempted to isolate *Moniliophthora perniciosa* from cacao branches with witches’ broom symptoms and fungal signs without success. Therefore, the *M. perniciosa* DNA was extracted from a basidiocarp collected from one of the commercial farms sampled for *M. roreri* isolations (Table S1). The remaining fungal strains used in these evaluations came from the EAFIT University culture collection, where they were also kept at −80 °C as 20% glycerol stocks (Table S1).

Fungal strains were activated at 30 °C in MEA plates and grown in Sabouraud broth cultures (Merck, NJ, USA) for 48 h at 200 rpm and 30 °C to produce mycelia for DNA extractions. The *M. roreri* MR2 strain was grown at 30 °C in MEA plates until sporulation to obtain *M. roreri* spores. Spores were washed off the MEA cultures with 0.05% tween 80 (Merk, NJ, USA) and their concentration was adjusted using a Neubauer chamber (Boeco, Germany) as required. The resulting spore suspensions were used for DNA extraction from spore suspensions and to inoculate spore traps.

The identity of the fungal strains and *M. perniciosa* DNA were confirmed by analysis of partial regions of the internal transcribed spacer (ITS) of the ribosomal DNA. The ITS fragments were PCR amplified with the ITS1 and ITS4 primers [21] (see PCR amplification). The PCR products were Sanger sequenced using the same primers (ITS1 and ITS4). Sequences were processed, and the taxonomic identity was assigned using the Basic Local Alignment Search Tool (BLAST) and the NCBI database in the platform Geneious Prime (version 2020.2.4, New Zealand) (https://www.geneious.com accessed on 1 December 2022).

### 2.2. DNA Extractions

We used two DNA extraction methodologies, one for extracting DNA from mycelia, and the other from spore suspensions, spore traps, and *M. perniciosa* basidiocarp. For the former, the mycelium was harvested from the Sabouraud broth cultures and homogenized using liquid nitrogen, a mortar, and a pestle. Then, the DNeasy PowerSoil kit (Qiagen, Germany) was used to extract DNA from the homogenized mycelium, following the manufacturer’s instructions. For the latter, a modified protocol published elsewhere was implemented [22]. For spores suspensions, 1 mL of *M. roreri* spore suspension containing 2.9 × 10^6^, 2.9 × 10^5^, or 2.9 × 10^4^ spores per ml was placed in 50-mL falcon tubes and centrifugated at 4500 rpm for 10 min to collect the spores. For *M. roreri* spores in spore traps, 1 mL of the same spore suspension was spread on spore traps, consisting of 2.5 cm × 6.5 cm sections of crystal-clear adhesive tape (Tesa, Switzerland) attached to microscopy slides with the sticky side facing outwards (Figure S1). Spore traps were air-dried, cut into 0.7 cm × 0.7 cm pieces to facilitate DNA extraction, and placed in 50-mL falcon tubes. For *M. perniciosa* basidiocarp, 100 mg of fresh-weight tissue was placed in 50 mL falcon tubes. Ballotini beads of 4- and 2-mm diameter (~3 mL) were added to the tubes, and samples were mechanically disrupted six times for 30 s using a vortex (Labnet S0200 Model VX-200 Vortex Mixer) at maximum speed, with submersion in liquid nitrogen between disruption cycles to avoid DNA degradation.

Five mL of lysis buffer, containing 100 mM Tris pH 8.0 (PanReac, Castellar del Vallès, Spain)**,** 3 M sodium chloride (NaCl) (ProtoKimica, Bogotá, Colombia), 3% (*P*/*V*) cetrimonium bromide (CTAB) (Amresco, OH, USA), 20 mM EDTA (PanReac), 1% (*P*/*V*) polyvinylpyrrolidone (PVP-40, molecular weight 40,000) (Amresco, OH, USA) and 1% (*V*/*V*) β-mercaptoethanol (Amresco, OH, USA), were added to the disrupted samples. Then, they were incubated at 65 °C for 1 h. During this incubation, samples went through disruption cycles of 10 s every 10 min to facilitate spore lysis. An equal volume of chloroform: isoamyl alcohol (24:1) (Sigma-Aldrich, MO, USA) was added and mixed by inversion. Samples were centrifuged for 10 min at 5000 g, and the DNA in the upper aqueous phase was transferred to new 50-mL falcon tubes. Here, ×0.1 volumes of 3 M sodium acetate pH 5.2 (Amresco, OH, USA) and ×0.66 volumes of cold isopropanol (ITW Reagents, IL, USA) were added and mixed by inversion. Tubes were incubated at ×20 °C overnight, and the DNA was precipitated by centrifugation at 15,000× *g* for 10 min. The DNA pellets were washed twice with 3 mL of 70% ethanol (Sigma-Aldrich, MO, USA) and air-dried. Then, they were resuspended in 50 µL of TE buffer (Bio Basic, Markham, ON, Canada) containing 0.05 mg/mL of RNase A (Thermo Fisher Scientific, Boston, MA, USA) and incubated at 37 °C for 30 min. The RNase A was deactivated at 65 °C for five min, and the DNA suspensions were kept at −20 °C until needed. 

The DNA concentration and quality were assessed using a NanoDrop 2000 (Thermo Fisher Scientific spectrophotometer, USA). The DNA integrity was evaluated by electrophoresis using five µL of the extracted DNA in agarose (Amresco, OH, USA) gels at 1%. Gels were run for 90 min at 70 V and visualized using an Enduro GDS gel visualizer (Labnet, NJ, USA). The DNA concentration was adjusted to 3 ng per µL to maintain consistency in the evaluations unless otherwise stated.

### 2.3. PCR Amplification

Primers in these evaluations targeted ITS regions of the ribosomal DNA, including generic ITS1 and ITS4 primers [23] and *M. roreri*-specific Mr_ITSF and Mr_ITSR primers (Table S2). For designing the Mr_ITSF and Mr_ITSR primers, we identified ITS regions conserved in *M. roreri* but no other fungi from the GeneBank, including the close relative *M. perniciosa* (Table S2). The alignment and primer design used the global alignment with free end gaps and the primer design functionalities of the Geneious prime application (version 2020.2.3). For conventional PCR amplification, 2 µL of the extracted DNA were used in 20 μL reactions of EconoTaq PLUS (Lucigen, WI, USA) with the generic (ITS1 and ITS4) or the specific (Mr_ITSF and Mr_ITSR) primers at 0.5 mM. Thirty-four amplification cycles were carried out in a BIO-RAD T100 Thermal Cycler (Bio-Rad, CA, USA) with an annealing temperature of 57 °C and an extension time of 1 min. The remaining conditions followed the manufacturer’s specifications. The PCR products were visually inspected by loading five µL of the amplification products into agarose gels at 1.2% that were run and visualized as before.

### 2.4. qPCR Optimization

To establish the best quantitative PCR (qPCR) conditions, we performed two preliminary evaluations, one assessing varying annealing temperatures and another primers concentrations. In these evaluations, the qPCR reactions consisted of 2 µL of the extracted DNA in 10 μL reactions of Universal IT SYBR Green supermix (Bio-Rad, CA, USA) with the Mr_ITSF and Mr_ITSR primers. The evaluations were repeated twice using DNA extracted from the mycelia of *M. roreri* strains MR1 and MR2 and three technical replicates per sample (Table S1). Three replicates of *M. perniciosa* basidiocarp DNA and H_2_O were also included as negative and non-template controls.

The primers’ concentration was set at 0.25 mM for the first evaluation assessing annealing temperatures. The amplification used the CFX96 real-time system (Bio-Rad, CA, USA) with a PCR program consisting of a denaturing step of 3 min at 98 °C followed by 33 cycles of 30 sec at 98 °C, 30 s at annealing temperatures ranging between 55 °C and 64 °C (Table S3), and 15 sec at 77 °C. The DNA was quantified at the end of each cycle, and dissociation curves followed the last cycle following the manufacturer’s specifications. The second evaluation assessing primers concentrations included concentrations between 0.2 mM and 0.4 mM (Table S3). These evaluations used the same qPCR reaction and amplifications program, but the annealing temperature was set at 62 °C, considering the results of the first evaluation.

For both evaluations, the mean and standard deviation (SD) of the cycle thresholds (Ct) were calculated considering the three technical replicates of the two repetitions (n = 6). Differences between Cts means were evaluated using anova followed by a Tukey’s range test with the *anova* and *emmeans* functions of the R libraries *stats* (version 4.0.4) and *emmeans* (version 1.8.0) [24,25]. As for the PCR products, qPCR products were visually inspected in agarose gels at 1.2%. Dissociation curves were also considered. A qPCR reaction was considered only when they had a single band in the gels and single-peak dissociation curves.

### 2.5. qPCR Characterization and Validation

The reactions and amplifications for the qPCR characterization and validation used primer concentrations of 0.3 mM and 37 cycles with an annealing temperature of 62 °C, considering the results of the qPCR optimization. The remaining factors were kept the same unless otherwise stated, including the two repetitions using three technical replicates per evaluation, which were analyzed as before, and including negative (*M. perniciosa* DNA) and non-template (H_2_O) controls. To assess the specificity of the qPCR, we used 6 ng (2 µL at 3 ng/µL) of DNA extracted from the mycelia of *M. roreri* and non-*M. roreri* strains and *M. perniciosa* basidiocarp (Table S1).

The qPCR efficiency was calculated using a standard-curve analysis [20,26]. Specifically, Ct values were estimated for serial dilutions (×1/10n) of the DNA extracted from the mycelia of *M. roreri* strain MR2 with concentrations between 0.0003 and 30 ng per μL. A linear model was used to correlate Ct values with the logarithm with base ten (log10) of the DNA concentrations, assuring that the model had no deviation from linearity, homogeneity of variance, or normality by visual inspection. The model estimates were used to determine the qPCR efficiency according to Equation (1).

(1) $$E=(10^{-\frac{1}{m}}-1)\times 100$$

*E* denotes the qPCR efficiency, and *m* is the slope of the linear model [20,26]. These analyses used the function *lm* of R library *stat* (version 4.0.4) [23], and the results were visualized using the *stat_poly_line* and *stat_poly_eq* functions of the R library *ggpmisc* (version 0.0.5) and the R library *ggplot2* (version 3.3.3) [27]. This evaluation was also repeated twice but used ten technical replicates per sample instead of three. Negative and non-template controls were also included in each qPCR. The dilution with 0.0003 ng of DNA per μL was excluded from the analysis since it was below the linear dynamic range of the qPCR [20,26].

The qPCR’s 95% detection limit (LOD) was calculated using the logit approach [26]. Specifically, the probability of detecting *M. roreri* in a sample containing *M. roreri* DNA, i.e., a true positive, was estimated for the same serial dilutions used for the qPCR efficiency estimation (DNA ng/μL between 0.0003 and 30). For this evaluation, a positive qPCR reaction was defined as a qPCR reaction yielding a Ct of at least 30 cycles since non-template and negative controls had Cts over 33. The probabilities of a true positive were fitted into a general linearized model (GLM) using the Logit link function, the binomial error family, and the log10 of the DNA ng (DNA ng between 0.0006 and 60) as the predictor. The GLM was used to estimate the ng of DNA associated with the probability of obtaining a true result 95% of the time (i.e., 95% LOD). This analysis used the *GLM* and *predict* functions of the R library *stat* (version 4.0.4) and the R library *ggplot2* (version 3.3.3) for visualization [24,27].

To assess whether the qPCR could detect and quantify DNA from *M. roreri* spores, we extracted DNA from spore suspensions and spore traps containing 2.9 × 10^6^, 2.9 × 10^5,^ and 2.9 × 10^4^ spores. The extracted DNA was used in qPCR reactions as before. In this case, the evaluations were repeated two times (i.e., two qPCR runs), including three biological replicates each with two technical replicates per sample (Table S1). These evaluations also included replicates of serial dilutions (×1/10n) of the DNA extracted from the mycelia of *M. roreri* strain MR2 with concentrations between 30 and 0.003 ng per μL, as standard curve, and replicates of *M. perniciosa* basidiocarp DNA and H_2_O as negative and non-template controls. The Cts of the serial dilutions of strain MR2 were linearly correlated with the log10 of the DNA concentrations. Then, the resulting linear model was used to estimate the DNA ng in the spore suspension and spore trap samples [28]. Then, DNA ng estimates were used to assess the inter and intra-qPCR run variability. This analysis used the *lm* and *predict* functions of the R library *stat* (version 4.0.4) [24].

### 2.6. Spore Trap Device

All the components of the spore traps devices were acquired from commercial sellers and assembled by the Investigación en Electromagnetismo Aplicado group of the EAFIT University. They included: (1) an Arduino UNO R3 system, (2) an AVR microcontroller, (3) a commercial IP67-ABS box (18 cm × 8 cm × 7 cm), (4) an L298N H-bridge motor driver, a 12 V DC 60 rpm geared motor, a Solar-Powered Systems CN3065, (5) a LiPo 3.7 V 6000 mAh battery, (6) a 1 W 5.5 V Seeed monocrystalline solar panel (170 mA), (7) a DS3231 real-time clock (RTC), (8) an I2C bidirectional bus, (9) SHT31 Sensirion temperature and humidity sensor, (10) an I2C bidirectional bus, and (11) an DM3AT micro-SD connector. The blades carrying the spore traps were custom-made by additive manufacturing using a 3D printer and polylactic acid.

## 3. Results

The initial step for developing a qPCR protocol specific to *M. roreri* was to design a pair of *M. roreri*-specific primers. We decided to focus on the ITS region of the ribosomal DNA, as sequences for this region are the most abundant for *M. roreri* and closely related fungi. Sequence alignment of fungal ITS revealed potential primer-binding sites that distinguished between *M. roreri* and other fungi, including the close relative *M. perniciosa*. Therefore, a pair of primers (Mr_ITSF and Mr_ITSR) was designed to target these regions. Several single-nucleotide polymorphisms (SNPs) were found between *M. roreri* and fungi from different genera in these primer-binding sites, but only a few between *M. roreri* and *M. perniciosa*. Despite their lower SNP number, the Mr_ITSF and Mr_ITSR primers likely distinguished between both *Moniliophthora* species since SNPs were located towards the primers’ 3′-end (Figure S2). Therefore, we decided to continue with this primers pair.

As expected, Mr_ITSF and Mr_ITSR primers distinguished *M. roreri* from other fungi, including *M. perniciosa,* in the conventional PCR assay. While the generic primers (ITS1 and ITS4) amplified nearly 550 bp ITS segments in all evaluated fungi (Figure S3, lanes 2–10), the Mr_ITSF and Mr_ITSR primers amplified about 120 bp ITS fragments only from *M. roreri* strains MR1 and MR2 (Figure S3, lanes 12–20). Therefore, we proceeded with the qPCR evaluations and the optimization of the qPCR conditions. In qPCR, Mr_ITSF and Mr_ITSR primers behaved best at concentrations of 0.3 mM and with an annealing temperature of 62 °C. Among the temperatures and concentrations evaluated, these conditions yielded the lowest Cts for the DNA suspensions of *M. roreri* strains MR1 and MR2 while yielding Cts above 33 for the non-template and negative controls (Table S2). They also generated a single peak around 80 °C in the dissociation curves (Figure S4A) and a single band in the agarose gel (Figure S4B). Considering the above, we decided to characterize and validate the *M. roreri* qPCR using the Mr_ITSF and Mr_ITSR primers at 0.3 mM, an annealing temperature of 62 °C, and 37 amplification cycles.

### 3.1. Moniliophthora roreri qPCR’s Specificity, Efficiency, Limit of Detection, and Presicion

The diversity of *M. roreri* in Colombia is high, and at least five populations have been identified [29,30]. To evaluate whether the qPCR could differentiate different *M. roreri* strains from other fungi, we used the DNA of *M. roreri* isolates from representative cacao-growing regions in Colombia. Ct varied widely between *M. roreri* isolates (nearly 9 cycles, from 15 to 24 cycles) (Table 1). This variation was unexpected since the qPCR reactions used comparable DNA amounts (6 ng). Despite the variation, the qPCR differentiated the *M. roreri* isolates from other fungi since all of them had higher Cts (>32 cycles, *p*-value < 0.05) (Table 1). Furthermore, strong 120 bp-bands were evident in the agarose gels only for the qPCR reactions of the *M. roreri* isolates (Figure S5). These results indicate that the qPCR can detect *M. roreri* isolates from several Colombian cacao-growing regions and distinguish them from other fungi, at least among the evaluated fungi.

**Figure 1:**
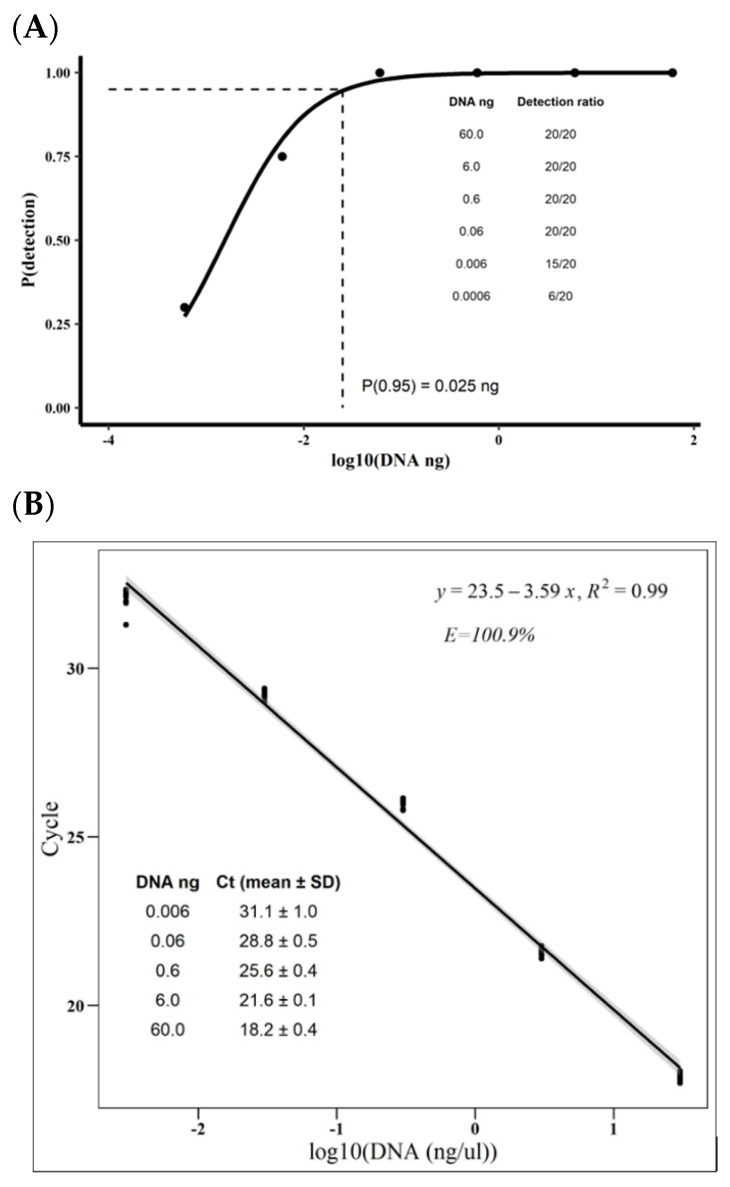
Limit of detection (LOD) and efficiency of the *Moniliophthora roreri* qPCR. Then, 2 μL of serial dilutions (×1/10n) of the DNA extracted from the mycelia of *M. roreri* strain MR2 with concentrations between 30 and 0.0003 ng per μL were evaluated in two separated qPCR, each containing ten technical replicates per dilution. Non-template (H_2_O) and negative (*M. perniciosa* basidiocarp DNA) controls were included in every qPCR. (**A**) shows the correlation between the probability of detecting the *M. roreri* DNA (i.e., return a positive qPCR) and the log10 of DNA amount in the serial dilutions (between 60 and 0.0006 ng). The points represent the probability of the *M. roreri* DNA dilutions to return a positive qPCR, defined as a qPCR reaction with a Ct below 30. The solid line represents the prediction of the general linearize model (GLM) with the logit function and the binomial family error (Table S4). The dashed line represents the qPCR’s 95% detection limit. (**B**) shows the correlation between the qPCR threshold cycle (Ct) and the logarithm with base 10 (log10) of the DNA concentration of the *M. roreri* serial dilutions. The points (n = 20 per log10 of DNA concentration) represent each sample. The line and the gray area represent the prediction and standard error of the linear model (lm), respectively.

Considering that the sensitivity and accuracy of the qPCR depend on the amount of the target in the qPCR reactions [20], we assessed the qPCR using different amounts of DNA of *M. roreri* strain MR2. We decided to use strain MR2 since it was among those with the higher Cts in the specificity evaluations (Table 1). The probability of the qPCR detecting *M. roreri* DNA (true positive) formed a sigmoid curve against the log10 of DNA ng, with the probability being 1 for over 0.06 ng of *M. roreri* DNA and closer to 0 as the ng of DNA dropped (Figure 1A). According to the glm correlating qPCR-detection probability with the log10 of the *M. roreri* DNA (Table S4), the qPCR’s 95% limit of detection (95% LOD) was 0.025 ng of *M. roreri* DNA (Figure 1 A), indicating that the qPCR should accurately detect *M. roreri* at least 95% of the time in samples with at least 0.025 ng of *M. roreri* DNA. The qPCR could detect fewer ng of DNA, but the risk of a false negative increased as the amount of DNA decreased. For example, the qPCR detected *M. roreri* DNA in 75% of the samples with 0.006 ng of *M. roreri* DNA. In contrast, it detected *M. roreri* DNA only in 30% of the samples containing 0.0006 ng of *M. roreri* DNA.

Regarding the accuracy and precision, the qPCR’s linearity range was between 0.006 and 60 ng of DNA, as indicated by the correlation between Ct values and the log10 of the serial dilutions of *M. roreri* DNA (R^2^: 0.98). In the linearity range, the Ct values lowered around 3.3 cycles for each ×10 increase in the DNA load, indicating a qPCR efficiency (E) of 100.9% (Figure 1B). DNA amounts below this range (0.0006 ng) compromised the lm fitting and the qPCR efficiency (R^2^: 0.96 and E: 115.4%) (Figure S6), showing that the reliability of the technique drops below this range. Consequently, the Ct variability was most pronounced at the lower limit of the qPCR linearity range (0.006 ng of DNA). For example, while 0.006 ng of DNA had a mean Ct of 31.1 with a standard deviation (SD) of 1, 0.6 had a mean Ct of 25.6 and an SD of 0.4 (Figure 1B).

The qPCR properly and consistently estimated the ng of *M. roreri* DNA in samples with amounts of DNA within the linearity range. The estimates for suspensions containing 6 and 0.6 ng of *M. roreri* DNA were 6.51 ± 0.27 ng and 0.80 ± 0.11 ng, showing coefficients of variation (CV) between and within qPCR runs below 14% (Table 2).

### 3.2. Moniliophthora roreri Spores Quantification

All of the above showed that the qPCR reliably detected at least 0.025 ng *M. roreri* DNA and could accurately quantify ng of *M. roreri* DNA between 0.006 and 60 ng. Now, we wondered if the qPCR was sensitive enough to detect and quantify DNA from *M. roreri* spore samples. For this purpose, we modified a previously published protocol [22] to extract DNA from spore suspension and spore traps containing 2.9 × 10^6^, 2.9 × 10^5,^ and 2.9 × 10^4^ *M. roreri* spores. Spore suspensions with 2.9 × 10^6^ and 2.9 × 10^5^ spores yield 7.5 ± 3.9 (CV = 52.0%) and 1.13 ± 0.24 (CV = 21.2%) μg of total DNA. On the other hand, spore traps with 2.9 × 10^6^ spores yield 0.13 ± 0.01 ug (CV = 7.7%) of total DNA, nearly ×1/58 lower than that of spore suspensions with the same spore load. The amount of DNA extracted from the remaining samples, including spore suspensions with 2.9 × 10^4^ spores, was too low to be quantified by NanoDrop. The results above showed that our protocol successfully extracted DNA from *M. roreri* spores in suspension and spore traps. However, the extraction was less efficient for spores in the spore traps and the yield was low for the samples with low spore loads.

Regarding the detection and quantification of DNA from *M. roreri* spore samples, all the estimates were above the 0.025 ng 95% LOD and within the 0.006 ng and 60 ng linear range of the qPCR. The exception was the estimates of spore suspensions with 2.9 × 10^6^ spores which were above the linearity range (Table 2). As expected, the estimated ng of DNA decreased with the spore loads and was higher (nearly ×10) for spore suspension than for spore traps. Overall, the estimates for spore suspensions with 2.9 × 10^6^, 2.9 × 10^5^, and 2.9 × 10^4^ spores were 81.83 ± 25.27, 10.9 ± 3.76, 0.56 ± 0.06 ng of estimated DNA compared with 8.36 ± 3.55, 0.35 ± 0.20, 0.07 ± 0.04 ng of estimated DNA for spore traps with the same spore loads (Table 2). Nonetheless, these estimates had a high variability compared with DNA suspensions, with the variation between the biological replicates in the same qPCR run (within-qPCR run CV) being particularly high. While the within-qPCR run CVs varied from 12% to 173% (vs. <13% for the DNA suspension estimates), the between-qPCR runs CVs from 10% to 57% (vs. <14% for the DNA suspension estimates) (Table 2). Despite this variation, these results showed that the qPCR can detect and quantify DNA from *M. roreri* spores in suspensions and spore traps. However, its variability must be considered when evaluating the *M. roreri* spore loads in the cacao fields.

### 3.3. Spore Trap Devices

This study aimed to develop a technique to evaluate the *M. roreri* spore loads in commercial cacao plantations. Therefore, we developed a homemade-spore-trap device to carry the spore traps for field evaluations. The spore-trap devices consist of an Arduino UNO R3 system with an AVR microcontroller encapsulated in a commercial IP67-ABS box of 18 cm × 8 cm × 7 cm. The Arduino UNO system has an L298N H-bridge motor driver connected to a PWM output, which controls a 12 V DC 60 rpm geared motor moving 20 cm-long blades carrying the spore traps (Figure S1). The spore-trap devices are powered by a Solar-Powered Systems CN3065 which is connected to a LiPo 3.7 V 6000 mAh battery and a 1 W 5.5 V Seeed monocrystalline solar panel (170 mA). Furthermore, they have a DS3231 real-time clock (RTC) coupled to the Arduino UNO R3 communicating via an I2C bidirectional bus. For collecting environmental data, the devices have SHT31 Sensirion temperature and humidity sensors attached to the I2C bidirectional bus. The data collected are stored in a 32 GB Sandisk memory connected to a DM3AT micro-SD connector and can be downloaded to a computer for its analysis.

The present study did not include the assessment of *M. roreri* spore loads in the fields. However, the spore-trap device can be used for this purpose since it was custom-made to carry the same spore traps used in the *Moniliophthora roreri* spores quantification. Further evaluations must validate the spore trap device in the cacao plantations

## 4. Discussion

Coupling spore traps with pathogen-specific qPCR is a reliable method for detecting and quantifying spores of fungal plant pathogens in the field [16,17,18,19] and could be useful for assessing the *M. roreri* spore loads in cacao plantations. Therefore, we developed a qPCR protocol to detect and quantify *M. roreri* DNA. According to our estimations, this method can distinguish *M. roreri* from other fungi and detect down to 0.025 ng of *M. roreri* DNA (95% LOD). It can also reliably quantify between 0.006 ng and 60 ng of *M. roreri* DNA.

While developing a qPCR protocol for detecting and quantifying spores of fungal plant pathogens, the specificity is crucial since air-born spores of several fungi, including plant pathogenic and non-pathogenic fungi, populate the crops environment [16,26,31,32]. Our qPCR protocol was successful in distinguishing *M. roreri* from other fungi, including its close relative, *M. perniciosa* [4]. Distinguishing between the *Moniliophthora* species is critical since *M. perniciosa* is also a cacao pathogen responsible for the Witches’ broom [33]. In Colombia, Witches’ broom (WBD) is a disease secondary to FPR, as its derived losses are lower [4]. However, in other countries such as Brazil, where FPR was only recently introduced, WBD is more relevant than FPR [3]. Failing to distinguish between *M. roreri* and *M. perniciosa* can lead to false positives and an overestimation of the *M. roreri* spore load.

Besides being specific, a method for assessing spore loads of plant pathogenic fungi must be sensitive and precise since spore loads are low in the field, meaning it must have low LOD and CV [26]. The 0.025 ng 95% LOD of our method suggests that it can reliably detect spore loads of at least 1.2 × 10^4^ spores, supposing mononucleated spores with a single ITS copy and a genome size close to 50 Mb [31,32]. However, the number of spores is probably lower since fungi have several copies of ribosomal DNA (between 14 and 1442 copies) [34], therefore ITS. The number of ribosomal DNA copies in *M. roreri* is unknown since, to our knowledge, there are no studies have addressed this issue. However, the LOD of our qPCR protocol is in line with similar evaluations in other systems [32,35].

Regarding the precision, the qPCR protocol yielded consistent estimates from *M. roreri* DNA suspensions (between and within qPCR runs CV close below 14%). However, the estimates for DNA extracted from *M. roreri* spores in suspension and spore traps were more variable (CV between 12% and 173%). The higher CVs for spore samples compared with DNA suspension are likely derived from the complexity of the biological sample. Extracting DNA from fungal spores is not trivial, especially from spore traps [26,28]. We used a modified protocol for extracting DNA from *M. roreri* spore samples trying to overcome some of the difficulties associated with the complexity of the samples [22], and despite being able to extract DNA from the spores, the yields varied from one sample to another and from one experiment to another. Despite the variation, the DNA estimates for most *roreri* spore samples were above the 95% LOD and within the linear range of the qPCR, meaning that our methods can detect and measure the DNA from spore suspension and spore traps containing at least 2.9 × 10^4^ *M. roreri* spores. Therefore, it could be used to assess the *M. roreri* spore loads in cacao plantations. However, the variability of the method must be considered and can be reduced by increasing the sample size.

We designed a spore-trap device to carry the spore traps, but this device still requires validation. Therefore, further evaluations must validate the spore trap device and the qPCR protocol in cacao plantations. We expect our method (coupling the spore trap device and the qPCR protocol) to be sensitive enough to assess the *M. roreri* spore loads in the field since evaluations using similar methods in other systems have reported between 1.0 and 1.0 × 10^5^ spores or DNA copies per m^3^ of air [18,19,36]. To our knowledge, no comparable analyses have been conducted for *M. roreri* in cacao fields. However, an older study using passive spore traps and microscopy detected between 30 and 144 *M. roreri* spores per cm^2^ of spore trap in an 8-h evaluation period [5]. These values are lower than those in our evaluations (i.e., 2.9 × 10^4^ *M. roreri* spores), and it is hard to predict if the sensitivity of our method will be enough to detect the *M. roreri* spore loads in the cacao plantations due to the methodology differences.

Even though our study was limited to laboratory evaluations, we consider that our method has great potential for estimating natural *M. roreri* spore loads in cacao crops. Following some validation, future work can use the spore-trap device and the *M. roreri*-specific qPCR protocol to estimate environmental *M. roreri* spore loads and evaluate how they are affected by the environment since the spore-trap device records climatic variables.

## Figures and Tables

**Table 1:** qPCR results for the evaluations assessing the specificity of the qPCR, including *Moniliophthora roreri* strains originated from several cacao regions in Colombia and other fungi.

Strain ^a^	Fungi	Ct (Mean ± SD) ^b^	Tukey Grouping ^c^	Isolation Site
MR 124	*Moniliophthora roreri*	15.9 ± 0.3	i	Necoclí, Antioquia
MR126		16.6 ± 0.5	hi	Necoclí, Antioquia
MR68		16.8 ± 0.1	hi	Palestina, Caldas
MR1		16.8 ± 0.3	hi	Barrancabermeja, Santander
MR134		17.0 ± 0.1	h	Orito, Putumayo
MR98		18.2 ± 0.1	g	Villanueva, Casanare
MR38		18.8 ± 0.2	fg	Barrancabermeja, Santander
MR136		19.8 ± 0.2	ef	Orito, Putumayo
MR33		19.8 ± 0.1	ef	San José del Nus, Antioquia
MR108		19.8 ± 0.3	ef	Rivera, Huila
MR30		19.9 ± 0.3	e	San José del Nus, Antioquia
MR28		21.1 ± 0.2	d	San José del Nus, Antioquia
MR2		21.1 ± 0.5	d	Barrancabermeja, Santander
MR48		23.1 ± 0.1	c	Paz de Ariporo, Casanare
EAFIT-F0059	*Alternaria argyroxiphii*	32.3 ± 0.8	b	--
--	*Pleurotus* sp.	32.8 ± 0.4	ab	--
N.A.	*Moniliophthora perniciosa*	33 ± 0.3	ab	Barrancabermeja, Santander
--	*Ganoderma* sp.	33.3 ± 0.2	ab	--
EAFIT-F0056	*Diaporthe phaseolorum*	33.3 ± 0.5	ab	--
EAFIT-F0066	*Colletotrichum siamense*	33.8 ± 0.5	a	--
N.A.	H_2_O	33.8 ± 0.2	a	--

^a^ Strain name acording to the universidad EAFIT culture collection; --, not avaliable; N.A., not aplicable. ^b^ Ct, qPCR threshold cycle; SD, standard deviation (n:6). ^c^ Same letters mean statistical grouping according to the Tukey test, 95% confidence leve (*p*-value < 0.05).

**Table 2:** qPCR estimates and within and between qPCR runs coefficients of variance for the *Moniliophthora roreri* DNA in suspension and extracted from *M. roreri* spores in suspension and spore-traps.

qPCR Run	Load	Within qPCR Run		Between qPCR Runs	
		Estimated DNA ng (Mean ± SD) ^a^	CV ^b^	Estimated DNA ng (Mean ± SD) ^c^	CV ^d^
DNA suspension					
1	6 ng	6.32 ± 0.59	0.09	6.51 ±0.27	0.04
2		6.7 ± 0.84	0.13		
1	0.6 ng	0.72 ± 0.08	0.11	0.80 ± 0.11	0.14
2		0.87 ± 0.02	0.02		
Spore suspensions					
1	2.9 × 10^6^ spores	99.7 ± 11.68	0.12	81.83 ± 25.27	0.31
2		63.96 ± 28.62	0.45		
1	2.9 × 10^5^ spores	13.56 ± 3.62	0.27	10.9 ± 3.76	0.34
2		8.25 ± 7.92	0.96		
1	2.9 × 10^4^ spores	0.52 ± 0.78	1.5	0.56 ± 0.06	0.1
2		0.6 ± 0.81	1.35		
Spore traps					
1	2.9 × 10^6^ spores	10.87 ± 6.51	0.6	8.36 ± 3.55	0.42
2		5.86 ± 6.64	1.13		
1	2.9 × 10^5^ spores	0.49 ± 0.62	1.26	0.35 ± 0.20	0.57
2		0.21 ± 0.16	0.78		
1	2.9 × 10^4^ spores	0.1 ± 0.16	1.73	0.07 ± 0.04	0.56
2		0.04 ± 0.01	0.14		
No-template control (H_2_O)					
1	--	0 ± 0	--	0 ± 0	--
2	--	0 ± 0	--		
Negative Control (M. pernisiosa DNA)					
1	--	0 ± 0	--	0 ± 0	--
2	--	0 ± 0	--		

^a^ Mean and SD, the standard deviation for the estimates of the biological replicates of each qPCR runs (n:3). ^b^ CV, Coeficient of variance (SD/mean) for the estimates of the biological replicates of each qPCR runs. ^c^ Mean and SD, the standard deviation for the estimates of the qPCR runs (n:2). ^d^ CV, Coefficient of variance (SD/mean) for the estimates of the biological replicates of each qPCR runs.

## Data Availability

The authors declare that the data supporting the findings of this study are available within the article.

## Supplementary Materials

The following supporting information can be downloaded at: https://www.mdpi.com/article/10.3390/jof9010047/s1; Figure S1. Spore-trap device developed for assessing the *Moniliophthora roreri* spore load in cacao plantations. (A) shows a picture of the device with a spore trap by itself and in the blade (detail). (B) is a block diagram showing the main components of the spore-trap device; Figure S2. Sequence alignment of the ITS sequences of the *Moniliophthora roreri* strains MR2 (OM056946) and MCA2954 (Genbank DQ222927) and *Moniliophthora perniciosa* strain COAD2616 (Genbank MK785158) and basidiocarp (Genbank OM056947) showing the binding sites of the Mr_ITSF and Mr_ITSR primers; Figure S3. Agarose gel showing the PCR products for the ITS fragments of *Moniliophthora roreri* and other fungi amplified with the ITS1 and ITSR4 (Lanes: 1–10) and the Mr_ITSF and Mr_ITSR primers (Lanes: 11–20). Lanes 1 and 11: 100 bp ladder. Lanes 2 and 12: *M. roreri* strain MR1. Lanes 3 and 13: *M. roreri* strain MR2. Lanes 4 and 14: *M. perniciosa* basidiocarp. Lanes 5 and 15: *Diaporthe* sp. EAFIT-F0056. Lanes 6 and 16: *Alternaria* sp. EAFIT-F0059. Lanes 7 and 17: *Colletotrichum* sp. EAFIT-F0066. Lanes 8 and 18: *Pleurotus* sp. Lanes 9 and 19: *Ganoderma* sp. Lanes 10 and 20: Non-template control; Figure S4. Representative images showing the dissociation curves (A) and agarose gel with the amplification products (B) of the qPCR targeting the ITS of *Moniliophthora roreri* with the Mr_ITSF and Mr_ITSR primers. The curves and gel belong to one of the experiments assessing different primers concentrations. In B, Lane 1: 100 bp ladder. Lane 2–5: 0.2 mM and 0.2 mM; Lane 6–9: 0.3 mM and 0.3 mM; and Lane 10–13: 0.4 mM and 0.3 mM (Mr_ITSF and Mr_ITSR primers concentration, respectively). Also, Lanes 2, 6, and 10: *M. roreri* strain MR1; Lanes 3, 7, and 11: *M. roreri* strain MR2; Lanes 4, 8, and 12: *M. perniciosa* basidiocarp; Lanes 5, 9, and 13: Non-template control; Figure S5. Agarose gel showing the qPCR products of the ITS fragments of *Moniliophthora roreri* and other fungi amplified with the Mr_ITSF and Mr_ITSR primers. Lanes 1 and 12: 100 bp ladder. Lanes 2: *M. roreri* strain MR1. Lane 3: *M. roreri* strain MR2. Lane 4: *M. perniciosa* basidiocarp. Lane 5: *Pleurotus* sp. Lane 6: *Ganoderma* sp. Lane 7: *Alternaria* sp. EAFIT-F0059. Lane 8: *Diaporthe* sp. EAFIT-F0056. Lane 9: *Colletotrichum* sp. EAFIT-F0066. Lane 10: *M. roreri* strain MR30. Lane 11: *M. roreri* strain MR33. Lane 13: *M. roreri* strain MR28. Lane 14: *M. roreri* strain MR48. Lane 15: *M. roreri* strain MR38. Lane 16: *M. roreri* strain MR98. Lane 17: *M. roreri* strain MR68. Lane 18: *M. roreri* strain MR108. Lane 19: *M. roreri* strain MR124. Lane 20: *M. roreri* strain MR126. Lane 21: *M. roreri* strain MR136. Lanes 22: Non-template control; Figure S6. Correlation between the qPCR threshold cycle (Ct) and the logarithm with base 10 (log10) of serial dilutions (×1/10n) of the DNA extracted from the mycelia of *M. roreri* strain MR2 with concentrations between 30 and 0.0003 ng per μL were evaluated in two separated qPCR, each containing ten technical replicates per dilution. Non-template (H2O) and negative (*M. perniciosa* basidiocarp DNA) controls were included in every qPCR. A) shows the correlation between the qPCR threshold cycle (Ct) and the log10 of the DNA concentration of the *M. roreri* serial dilutions. The points (n = 20 per log10 of DNA concentration) represent each sample. The line and the gray area represent the prediction and standard error of the linear model (lm), respectively; Table S1. Fungal strains used in this study; Table S2. Primers and fungal ITS sequences used in this study; Table S3. qPCR results for the evaluations assessing melting different temperatures and primers concentrations; Table S4. Estimates for the general linearized model (glm) with the logit function and the binomial family error correlating thedetection probability of the qPCR with the logarithm with base 10 (log10) of *Moniliophthora roreri* DNA in serial dilutions.
