# Supplementary material for: Development of a Method for Detecting and Estimating Moniliophthora roreri Spore Loads Based on Spore Traps and qPCR
Source: J Fungi (Basel). 2022 Dec 28;9(1):47. doi: 10.3390/jof9010047 (PMC9862605; doi:10.3390/jof9010047)
Supplement: Supplementary file 1 [file jof-09-00047-s001.zip › jof-1950436-supplementary.pdf]

Journal of *Fungi*

MDPI

A

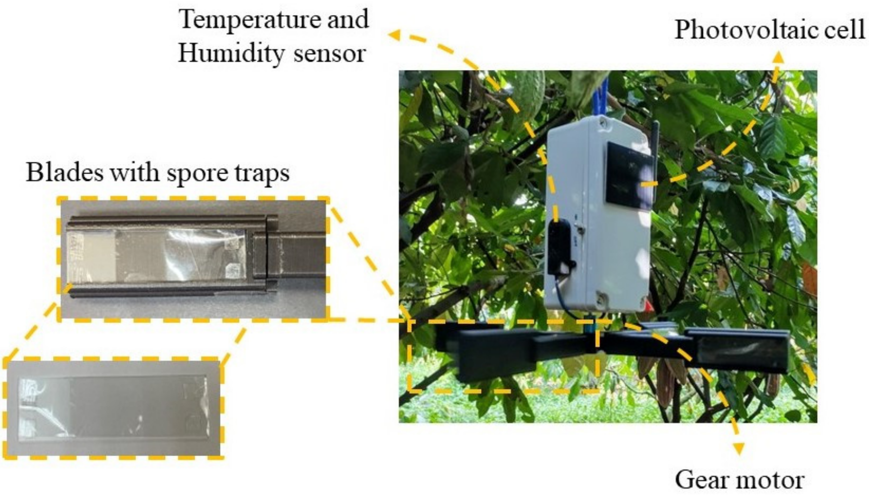

B

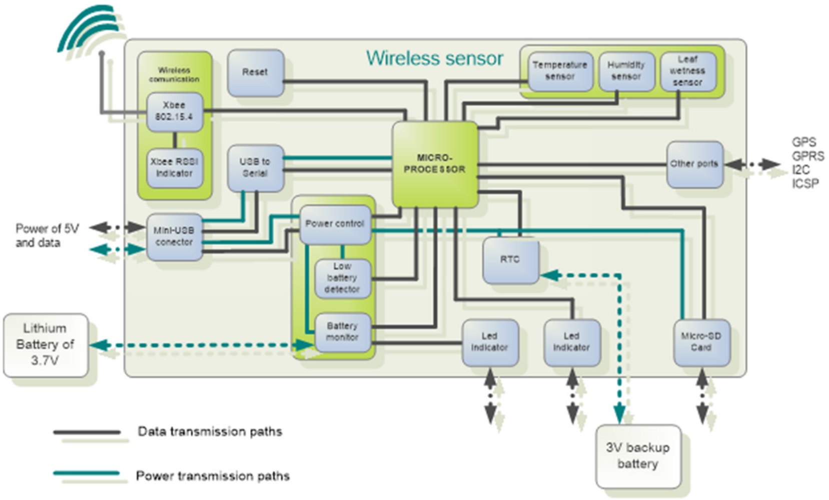

Figure S1. Spore-trap device developed for assessing the *Moniliophthora roreri* spore load in cacao plantations. A) shows a picture of the device with a spore trap by itself and in the blade (detail). B) is a block diagram showing the main components of the spore-trap device.

```
M. roreri MR2             ACCAAGAGGCGCAAGGTGCGTTCAAAGATTCGATGATTCACTGAATTCTGCAATTCACATTACTTATCGCATTTC  414
M. roreri MCA2954         ACCAAGAGGCGCAAGGTGCGTTCAAAGATTCGATGATTCACTGAATTCTGCAATTCACATTACTTATCGCATTTC  402
M. pernisiosa COAD2616    ACCAAGAGGCGCAAGGTGCGTTCAAAGATTCGATGATTCACTGAATTCTGCAATTCACATTACTTATCGCATTTC  420
M. perniciosa basidiocarp ACCAAGAGGCGCAAGGTGCGTTCAAAGATTCGATGATTCACTGAATTCTGCAATTCACATTACTTATCGCATTTC  385

                                                                               Mr_ITSF:   GGTCAATTAAG
M. roreri MR2             GCTGCGTTCTTCATCGATGCGAGAGCCAAGAGATCCGTTGCTGAAAGTTGTATAGTTTTTAAAGGGTCAATTAAG  489
M. roreri MCA2954         GCTGCGTTCTTCATCGATGCGAGAGCCAAGAGATCCGTTGCTGAAAGTTGTATAGTTTTTAAAGGGTCAATTAAG  477
M. pernisiosa COAD2616    GCTGCGTTCTTCATCGATGCGAGAGCCAAGAGATCCGTTGCTGAAAGTTGTATAGTT-TTAAAGGGTCAATTAAG  494
M. perniciosa basidiocarp GCTGCGTTCTTCATCGATGCGAGAGCCAAGAGATCCGTTGCTGAAAGTTGTATAGTT-TTAAAGGGTCAATTAAG  459

Consensus                 TCCCAATAAAATACACA
M. roreri MR2             TCCCAATAAAATACACATTCTAAACATACATTAAGTGTGTGTTA-A---A-ACATAGTGAGACTCCGTACTGAGA  559
M. roreri MCA2954         TCCCAATAAAATACACATTCTAAACATACATTAAGTGTGTGTTA-A---A-ACATAGTGAGACTCCGTACTGAGA  547
M. pernisiosa COAD2616    TCCCAATAAAATAC--ATTCTAAACATACATTAAGTGTGT-T.AAAAACAAACATAGT-AGACTCCGTACT.AGA  565
M. perniciosa basidiocarp TCCCAATAAAATAC--ATTCTAAACATACATTAAGTGTGT-T.AAAAACAAACATAGT-AGACTCCGTACT.AGA  530

                               Mr_ITSR:   CC-GTTCCAGAATCCACTACAAAAG
M. roreri MR2             -AAACGCAAGCGCCTCCC-GTTCCAGAATCCACTACAAAAGGTTCACAGGTGGATGAAGATTGAAAGTCGGCGAG  632
M. roreri MCA2954         -AAACGCAAGCGCCTCCCCGTTCCAGAATCCACTACAAAAGGTTCACAGGTGGATGAAGATTGAAAGTCGGCGAG  621
M. pernisiosa COAD2616    .AAACGCAAGCGCCTCCC..TTCCAGAATC..CTACAAAAGGTTCACAGGTGGATGAA..TTGAAAGTCGGCGAG  640
M. perniciosa basidiocarp .AAACGCAAGCGCCTCCC..TTCCAGAATC..CTACAAAAGGTTCACAGGTGGATGAA..TTGAAAGTCGGCGAG  605


M. roreri MR2             CACATGCCCCTAAGAAGAGCCAGCTCAAAACCTC-----------------------------------------  707
M. roreri MCA2954         CACATGCCCCTAAGAAGAGCCAGCTCAAAACCTCCTTTACAATGTTTTCAATAATGATCCTTCCG----------  696
M. pernisiosa COAD2616    CACATGCC-CTAC-AAGAGCCAGCTCAA--CCTCCTTTACAATGTTTTCAATAATGATCCTTCCGCAGGTTCACC  711
M. perniciosa basidiocarp CACATGCC-CTAC-AAGAGCCAGCTCA----------------------------------------------  678
```

Figure S2. Sequence alignment of the ITS sequences of the *Moniliophthora roreri* strains MR2 (OM056946) and MCA2954 (Genbank DQ222927) and *Moniliophthora perniciosa* strain COAD2616 (Genbank MK785158) and basidiocarp (Genbank OM056947) showing the binding sites of the Mr_ITSF and Mr_ITSR primers.

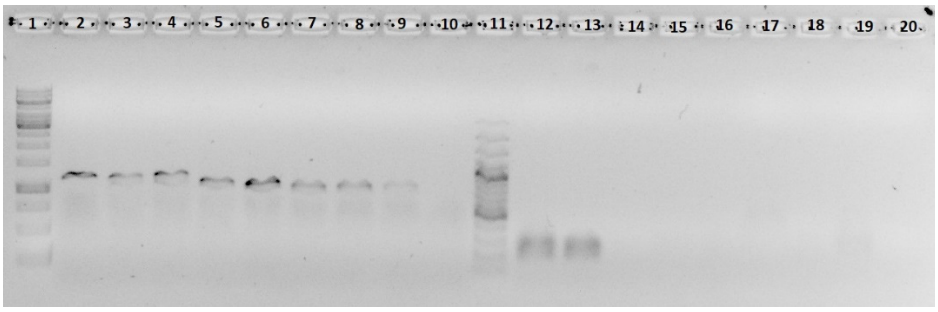

Figure S3. Agarose gel showing the PCR products for the ITS fragments of *Moniliophthora roreri* and other fungi amplified with the ITS1 and ITSR4 (Lanes: 1-10) and the Mr_ITSF and Mr_ITSR primers (Lanes: 11-20). Lanes 1 and 11: 100 bp ladder. Lanes 2 and 12: *M. roreri* strain MR1. Lanes 3 and 13: *M. roreri* strain MR2. Lanes 4 and 14: *M. perniciosa* basidiocarp. Lanes 5 and 15: *Diaporthe* sp. EAFIT-F0056. Lanes 6 and 16: *Alternaria* sp. EAFIT-F0059. Lanes 7 and 17: *Colletotrichum* sp. EAFIT-F0066. Lanes 8 and 18: *Pleurotus* sp. Lanes 9 and 19: *Ganoderma* sp. Lanes 10 and 20: Non-template control.

A

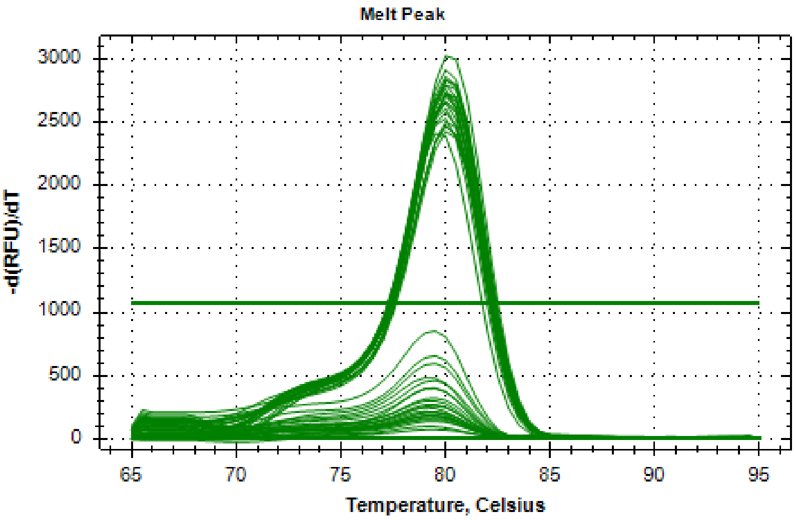

B

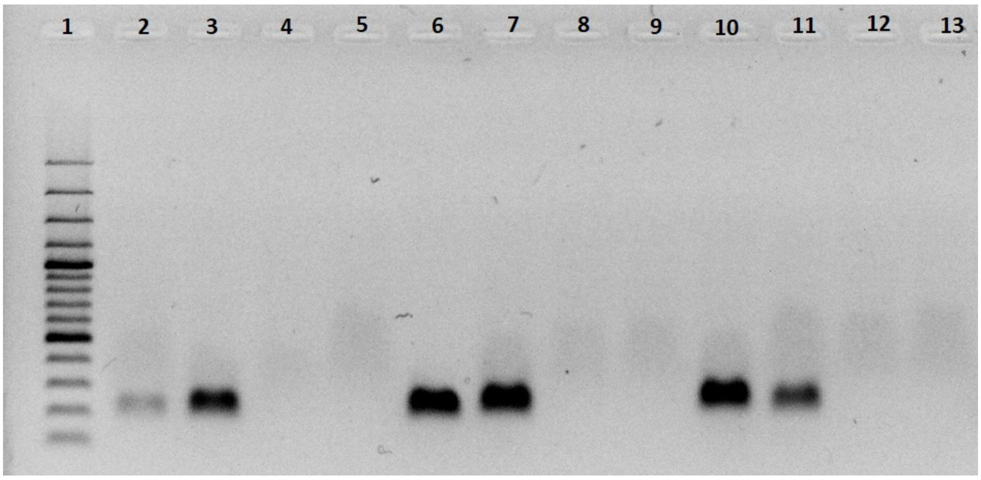

Figure S4. Representative images showing the dissociation curves (A) and agarose gel with the amplification products (B) of the qPCR targeting the ITS of *Moniliophthora roreri* with the Mr_ITSF and Mr_ITSR primers. The curves and gel belong to one of the experiments assessing different primers concentrations. In B, Lane 1: 100 bp ladder. Lane 2-5: 0.2 mM and 0.2 mM; Lane 6-9: 0.3 mM and 0.3 mM; and Lane 10-13: 0.4 mM and 0.3 mM (Mr_ITSF and Mr_ITSR primers concentration, respectively). Also, Lanes 2, 6, and 10: *M. roreri* strain MR1; Lanes 3, 7, and 11: *M. roreri* strain MR2; Lanes 4, 8, and 12: *M. perniciosa* basidiocarp; Lanes 5, 9, and 13: Non-template control.

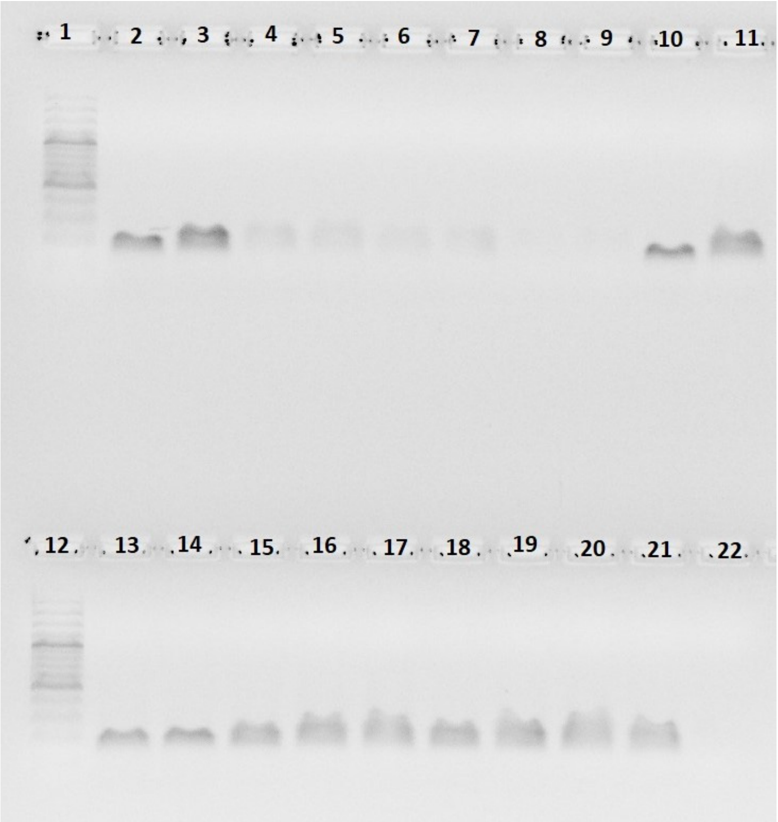

Figure S5. Agarose gel showing the qPCR products of the ITS fragments of *Moniliophthora roreri* and other fungi amplified with the Mr_ITSF and Mr_ITSR primers. Lanes 1 and 12: 100 bp ladder. Lanes 2: *M. roreri* strain MR1. Lane 3: *M. roreri* strain MR2. Lane 4: *M. perniciosa* basidiocarp. Lane 5: *Pleurotus* sp. Lane 6: *Ganoderma* sp. Lane 7: *Alternaria* sp. EAFIT-F0059. Lane 8: *Diaporthe* sp. EAFIT-F0056. Lane 9: *Colletotrichum* sp. EAFIT-F0066. Lane 10: *M. roreri* strain MR30. Lane 11: *M. roreri* strain MR33. Lane 13: *M. roreri* strain MR28. Lane 14: *M. roreri* strain MR48. Lane 15: *M. roreri* strain MR38. Lane 16: *M. roreri* strain MR98. Lane 17: *M. roreri* strain MR68. Lane 18: *M. roreri* strain MR108. Lane 19: *M. roreri* strain MR124. Lane 20: *M. roreri* strain MR126. Lane 21: *M. roreri* strain MR136. Lanes 22: Non-template control.

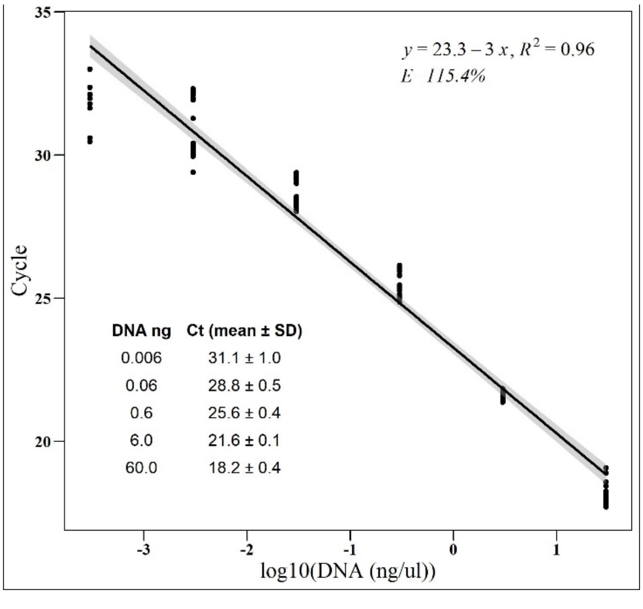

Figure S6. Correlation between the qPCR threshold cycle (Ct) and the logarithm with base 10 (log10) of serial dilutions (x 1/10n) of the DNA extracted from the mycelia of M.

roreri strain MR2 with concentrations between 30 and 0.0003 ng per µl were evaluated in two separated qPCR, each containing ten technical replicates per dilution. Non-template (H2O) and negative (*M. perniciosa* basidiocarp DNA) controls were included in every qPCR. A) shows the correlation between the qPCR threshold cycle (Ct) and the log10 of the DNA concentration of the *M. roreri* serial dilutions. The points (n = 20 per log10 of DNA concentration) represent each sample. The line and the gray area represent the prediction and standard error of the linear model (lm), respectively.

Table S1. Fungal strains used in this study.

	**Molecular identity**			
**Strain**	**Closest sequence**[a]	**GB AN**[b]	**HI**[c]	**Isolation site**
**Isolated during the study**				
MR1	*Moniliophthora roreri*	MH861051	100	Barrancabermeja, Colombia
MR2	*Moniliophthora roreri*	KU674835	100	Barrancabermeja, Colombia
MR124	*Moniliophthora roreri*	OP807173	100	Necoclí, Antioquia, Colombia
MR126	*Moniliophthora roreri*	OP820440	100	Necoclí, Antioquia, Colombia
MR68	*Moniliophthora roreri*	OP820441	100	Palestina, Caldas, Colombia
MR134	*Moniliophthora roreri*	OP807112	100	Orito, Putumayo, Colombia
MR98	*Moniliophthora roreri*	OP820439	100	Villanueva, Casanare, Colombia
MR38	*Moniliophthora roreri*	OP784768	100	Barrancabermeja, Santander, Colombia
MR136	*Moniliophthora roreri*	OP806971	100	Orito, Putumayo, Colombia
MR33	*Moniliophthora roreri*	OP784771	100	San José del Nus, Antioquia, Colombia
MR108	*Moniliophthora roreri*	OP820443	100	Rivera, Huila, Colombia
MR30	*Moniliophthora roreri*	OP781680	100	San José del Nus, Antioquia, Colombia
MR28	*Moniliophthora roreri*	OP781654	100	San José del Nus, Antioquia, Colombia
MR48	*Moniliophthora roreri*	OP820438	100	Paz de Ariporo, Casanare, Colombia
ND	*Moniliophthora perniciosa*	MH861049	100	Barrancabermeja, Colombia
**Culture collection Universidad EAFIT**				
ND	*Pleurotus* sp.	MK673812	100	--
ND	*Ganoderma* sp.	un-published	ND	--
EAFIT-F0056	*Diaporthe phaseolorum*	MN997107	99.8	--
EAFIT-F0059	*Alternaria argyroxiphii*	NR136074	100	--
EAFIT-F0066	*Colletotrichum siamense*	MZ066745	99.1	--

[a] Fungal strain with the highest ITS sequence similarity according to BLAST.
[b] GeneBank accession number.
[c] Highest Identity, values between 0 and 100 %. ND, not defined.

Table S2. Primers and fungal ITS sequences used in this study.

Primer	Sequence	Target	Reference
ITS1	TCCGTAGGTGAACCTGCGG	Fungal ITS	[1]
ITS4	TCCTCCGCTTATTGATATGC	Fungal ITS	[1]
Mr-ITSF	GGTCAATTAAGTCCCAATAAAATACACA	*M. roreri* ITS	This study
Mr-ITSR	CTTTTGTAGTGGATTCTGGAACGG	*M. roreri* ITS	This study
**ITS GB AN[b]**	**Fungal specie**	**Fungal strain**	**Reference**
OM056945[d]	*Moniliophthora roreri*	MR1	This study
OM056946	*Moniliophthora roreri*	MR2	This study
JX515287	*Moniliophthora roreri*	B1b	Unpublish
JX515288	*Moniliophthora roreri*	B2a	Unpublish
JX315285	*Moniliophthora roreri*	E16	Unpublish
JX315273	*Moniliophthora roreri*	SPCL	Unpublish
MH861051	*Moniliophthora roreri*	CBS 202.77	[2]
KU674835	*Moniliophthora roreri*	CBS138635	[3]
JX315282	*Moniliophthora roreri*	Co12	Unpublish
JX515290	*Moniliophthora roreri*	B3	Unpublish
JX515291	*Moniliophthora roreri*	B4	Unpublish
AY230254	*Moniliophthora roreri*	ND[e]	[4]
OM056947	*Moniliophthora perniciosa*	**ND**	This study
MK785162	*Moniliophthora perniciosa*	RWB1268	Unpublish
MK785163	*Moniliophthora perniciosa*	DIS70	Unpublish
MK785161	*Moniliophthora perniciosa*	RWB1267	Unpublish
MK785160	*Moniliophthora perniciosa*	RWB1205	Unpublish
MK785159	*Moniliophthora perniciosa*	RWB1065	Unpublish
MK785158	*Moniliophthora perniciosa*	COAD2616	Unpublish
MK785157	*Moniliophthora perniciosa*	COAD2615	Unpublish
MK785142	*Moniliophthora perniciosa*	COAS2600	Unpublish
MK785141	*Moniliophthora perniciosa*	COAS2599	Unpublish
MK785140	*Moniliophthora perniciosa*	COAD2598	Unpublish
MK785139	*Moniliophthora perniciosa*	COAD540	Unpublish
KY081771	*Moniliophthora perniciosa*	WMA14(B)	[5]
KY081770	*Moniliophthora perniciosa*	WMA5	[5]
KY081769	*Moniliophthora perniciosa*	SCFT	[5]
KY081768	*Moniliophthora perniciosa*	LJ8	[5]
EU514248	*Pseudocercospora fijiensis*	CBS 120258	[6]
AY616686	*Entonaema liquescens*	agtS279	[7]
KF225610	*Cytospora atrocirrhata*	HMBF156	Unpublish
JQ005152	*Colletotrichum gloeosporioides*	CBS 112999	[8]

JQ005776	*Colletotrichum acutatum*	CBS 112996	[9]
KJ909769	*Bipolaris maydis*	CBS136.29	Unpublish
AM749934	*Daldinia caldariorum*	JPP 26211	[10]
FJ889444	*Diaporthe alleghaniensis*	CBS 495.72	[11]
AF388914	*Neurospora crassa*	FGSC 987	[12]
KX986055	*Nigrospora oryzae*	LC6760	[13]
FJ889450	*Phomopsis cotoneastri*	CBS 439.82	[11]

[b] GeneBank accession number.

[c] Highest Identity, values between 0 and 100 %. ND not defined.

[d]Bold sequences are the sequences submitted to the GeneBank during this study.

[e]ND, no defined.

Table S3. qPCR results for the evaluations assessing melting different temperatures and primers concentrations.

Factor		*M. roreri* strain		Controls[a]	
		MR1	MR2	NTC	Negative
**Temperature (°C)**			Ct (mean ± SD)[b]		
	55.5	23.8 ± 0.2 (b)[c]	23.2 ± 0.1 (ab)	> 33	> 33
	56.5	24 ± 0.5 (b)	23.1 ± 1.2 (ab)		
	59.4	24.2 ± 0.3 (b)	23.1 ± 0.8 (ab)		
	**61.6**	**22.3 ± 0.9 (a)**	**21.8 ± 0.7 (a)**		
	62.7	24.1 ± 0.0 (b)	23.8 ± 0.1 (ab)		
	64	24 ± 0.0 (b)	24 ± 0.1 (b)		
**Primers' concentration (mM)[d]**			Ct (mean ± SD)		
	0.2 - 0.2	26.3 ± 0.1 (a)	25.6 ± 0.1 (bcd)	> 33	> 33
	0.2 - 0.3	26.2 ± 0.3 (a)	25.5 ± 0.1 (abc)		
	0.2 - 0.4	26.4 ± 0.2 (ab)	25.3 ± 0.1 (a)		
	0.3 - 0.2	26.9 ± 0.1 (b)	25.8 ± 0.1 (cde)		
	**0.3 - 0.3**	**26 ± 0.2 (a)**	**25.4 ± 0.1 (ab)**		
	0.3 - 0.4	26.2 ± 0.1 (a)	25.5 ± 0.1 (abc)		
	0.4 - 0.2	26.3 ± 0.1 (a)	25.7 ± 0.1 (bcd)		
	0.4 - 0.3	26 ± 0.2 (a)	25.9 ± 0.3 (de)		
	0.4 - 0.4	26.2 ± 0.1 (a)	26.1 ± 0 (e)		

[a] NTC: non-template control (H_2O); Negtive: Negative control (*M. perniciosa* basidiocarp)

[b] Ct, qPCR threshold cycle; SD, standard deviation (n : 6)

[c] Statistical grouping according to the Tukey test, 95% confidence leve (p-value < 0.05)

[d] Mr-ITSF and Mr-ITSR concentrations (Mr-ITSF - Mr-ITSR)

Table S4. Estimates for the general linearized model (glm) with the logit function and the binomial family error correlating the detection probability of the qPCR with the logarithm with base 10 (log10) of *Monili-ophthora roreri* DNA in serial dilutions.

model: glm (Probability ~ log10(DNA), family = binomial)				
	**Estimates**	**St. Error**	**Z value**	**p-value**
Intercept	6.68	1.57	4.27	<0.001
log10(spores)	2.38	0.57	4.17	<0.001
**Observations**				6
**Null deviance**				57.9
**Residual deviance**				1.4
**AIC**				11.89
Null model: glm (Probability ~ 1, family = binomial)				
**Fixed effects**				
	**Odds Ratios**	**St. Error**	**Z value**	**p-value**
Intercept	1.67	0.25	6.68	<0.001
**Observations**				6
**Null deviance**				57.9
**Residual deviance**				57.9
**AIC**				66.43
